# 1700 nm optical coherence microscopy enables minimally invasive, label-free, in vivo optical biopsy deep in the mouse brain

**DOI:** 10.1038/s41377-021-00586-7

**Published:** 2021-07-14

**Authors:** Jun Zhu, Hercules Rezende Freitas, Izumi Maezawa, Lee-way Jin, Vivek J. Srinivasan

**Affiliations:** 1 Department of Biomedical Engineering, University of California Davis, Davis, CA 95616 USA; 2 Department of Pathology and Laboratory Medicine, University of California Davis Medical Center, Sacramento, CA 95817 USA; 3 Department of Ophthalmology and Vision Science, School of Medicine, University of California Davis, Sacramento, CA 95817 USA; 4 Department of Ophthalmology, NYU Langone Health, New York, NY 10017 USA; 5 Department of Radiology, NYU Langone Health, New York, NY 10016 USA; 6 Tech4Health Institute, NYU Langone Health, New York, NY 10010 USA

**Keywords:** Biophotonics, Interference microscopy, Imaging and sensing

## Abstract

In vivo, minimally invasive microscopy in deep cortical and sub-cortical regions of the mouse brain has been challenging. To address this challenge, we present an in vivo high numerical aperture optical coherence microscopy (OCM) approach that fully utilizes the water absorption window around 1700 nm, where ballistic attenuation in the brain is minimized. Key issues, including detector noise, excess light source noise, chromatic dispersion, and the resolution-speckle tradeoff, are analyzed and optimized. Imaging through a thinned-skull preparation that preserves intracranial space, we present volumetric imaging of cytoarchitecture and myeloarchitecture across the entire depth of the mouse neocortex, and some sub-cortical regions. In an Alzheimer’s disease model, we report that findings in superficial and deep cortical layers diverge, highlighting the importance of deep optical biopsy. Compared to other microscopic techniques, our 1700 nm OCM approach achieves a unique combination of intrinsic contrast, minimal invasiveness, and high resolution for deep brain imaging.

## Abstract (web summary)

1700 nm optical coherence microscopy images neurons and axonal myelination across the mouse neocortex and some sub-cortical regions, based on intrinsic tissue contrast, with minimal invasiveness.

## Graphical abstract

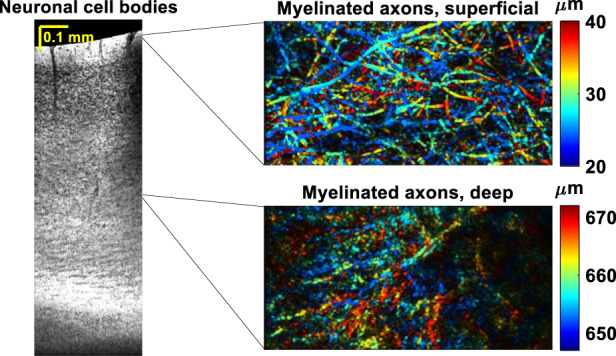

## Introduction

Central nervous system (CNS) diseases such as Alzheimer’s disease (AD) manifest early at the microscopic (i.e., cellular) level^1,2^ deep in the brain. However, high-resolution, in vivo brain imaging has been a long-standing challenge. Macroscale imaging techniques such as magnetic resonance imaging (MRI) and positron emission tomography (PET) enable structural and functional imaging at the whole brain level^3,4^. However, achievable PET resolution is limited to the millimeter or sub-millimeter scale^5,6^, and cellular resolution is not yet feasible in MRI^7^. Optical methods such as multi-photon microscopy (MPM) can achieve micron-scale resolution^1,8^, which facilitates cellular imaging, but usually require exogenous fluorescence labeling or transgenic models. Additionally, the signal-to-background ratio of MPM degrades in deep tissue. Imaging of deep cortical layers with two-photon microscopy, even via invasive cranial windows, is challenging^9^. Three-photon microscopy improves upon the signal-to-background ratio of two-photon microscopy^10^ and provides sub-cortical imaging through invasive cranial windows^11^ and mid-cortical imaging through the intact skull^12^. Optical coherence microscopy (OCM), which is a label-free imaging technology based on backscattered light, images cellular architecture up to layer V in the rodent brain at 1300 nm^13^ through an invasive cranial window. Yet, invasive preparations that remove overlying turbid tissue such as the skull and dura enable imaging deeper, but significantly perturb brain physiology^14,15^. The goal of cellular-level imaging in the deep cortical layers and beyond, through a minimally invasive preparation, remains elusive.

With this goal in mind, here we design and demonstrate the first OCM system for in vivo imaging of brain cellular architecture in the 1700 nm optical window, where ballistic attenuation in the brain is minimal^11,16–19^. Technical challenges of this emerging wavelength range are addressed through the system design, including the choice of light source, dispersion compensation method, and optical components. Coherence gating is shown to complement confocal gating^20^, while post-processing is optimized to reduce speckle while also rejecting multiply scattered light. Cytoarchitecture and myeloarchitecture are imaged across the neocortex and some sub-cortical regions, through a minimally invasive, thinned-skull surgical preparation. Laminar variations in neuropathology are investigated in a mouse model of Alzheimer’s disease, with corresponding histology for comparison. Amongst approaches to study neuropathology in the living, intact brain, 1700 nm OCM provides a unique combination of label-free, minimally invasive, deep, and high-resolution imaging.

## Results

**Fig. 1:**
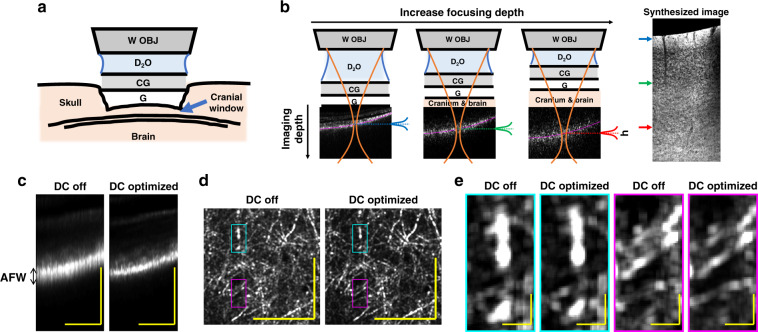
Minimally invasive 1700 nm optical coherence microscopy (OCM) with optimized dispersion compensation. **a** Schematic representation of the thinned-skull, glass coverslip-reinforced imaging preparation. W OBJ: water immersion objective, D_2_O: heavy water immersion medium, CG: cover glass, G: glue. **b** At each focal depth, volumetric data are acquired (example corresponding cross-sectional images shown). As depicted in Eq. (5), a weighting function, *h*, is applied to each OCM data set to synthesize an image volume (minimum intensity sagittal image shown). Note that as the sample is translated towards the objective, cranium and brain replace D_2_O along the light path. **c** The apparent focal width (AFW) in cross-section is found to be narrowed by numerical dispersion compensation (DC). A method of dispersion optimization based on this observation was devised (Supplementary Fig. S1) and validated (Supplementary Fig. S2). Optimized dispersion compensation results in sharper myelin features in layer I *en face* maximum intensity projections (**d**, **e**). Notably, the dispersion optimization method was found to work even when OCM volumes did not contain clear image features, enabling optimization of dispersion compensation at each focal depth (Supplementary Fig. S1). Scalebars represent 0.1 mm and 10 μm in **c**, **d** and **e**, respectively

To achieve three-dimensional and minimally invasive optical biopsy, OCM volumes were acquired through the skull, which was lightly thinned to ~50 μm to minimize potential damage to the cerebral cortex. A coverglass was affixed to the skull with superglue and heavy water (D_2_O) was employed as the immersion medium for the objective (Fig. 1a). The measured OCM transverse resolution was sub-micron, while the nominal coherence-gated axial resolution was 5.6 μm in tissue, and axial sectioning was further aided by the confocal gate (see Materials and Methods). Dynamic focusing was achieved by translating the sample towards the objective in 5 μm intervals (Fig. 1b). An OCM volume with a narrow depth-of-field, leading to a narrow range of imaging depths in Fig. 1b, was acquired at each focal position, yielding a 4-dimensional data set. To synthesize these data into a single volume while suppressing out-of-focus and multiply scattered light, an axial weighting function, *h*, was employed. Further details on the optimization of system, imaging parameters, and post-processing can be found in Materials and Methods.

### Optimization of dispersion compensation in the 1700 nm optical window

Chromatic dispersion must be compensated, either physically or numerically, to maintain optimal axial resolution in OCM^21^. As the focus is translated deeper into the brain, brain tissue [mostly water (H_2_O)^22^] replaces the D_2_O immersion medium along the sample optical path (Fig. 1b). This possibly leads to a focal depth-dependent chromatic dispersion; however, chromatic dispersion measurements of H_2_O and D_2_O in the 1700 nm window are sparse in the literature^23–25^, making this possibility challenging to assess. At the same time, conventional empirical image-based numerical dispersion optimization methods^26^ are not always applicable for shallow depth-of-field OCM images which lack clear features to aid optimization, particularly in deep tissue.

These limitations led us to develop a simple and robust way of optimizing numerical dispersion compensation for in vivo OCM (see Supplementary Section S1). We first noticed that the shallow depth-of-field of the OCM system confines the path length distribution of detected light (Fig. 1c–e). Then, to optimize numerical dispersion compensation, we sought to minimize the apparent focal width, defined as the axial width of the OCM intensity distribution, at each focal depth (Fig. 1c, Supplementary Fig. S1b, c). As this approach relies on the path length profile caused by tight light focusing, it is valid even if raw OCM images lack well-defined structures. As shown in Supplementary Fig. S1d–f, the optimal second-order dispersion compensation coefficient increased about 507 fs^2^ as the focus shifted from the brain surface to 900 μm depth, though systematic changes in the third-order coefficient were undetectable. Furthermore, even though the apparent focal width increased with deep focusing due to multiple scattering (Fig. 1b, Supplementary Fig. S1d), the method still provided consistent results.

To validate this method, in view of the previously-noted dearth of chromatic dispersion data in the 1700 nm wavelength range, we undertook our own measurements of H_2_O and D_2_O dispersion (Supplementary Fig. S2). We found that dispersion changes with focal depth, derived by our empirical in vivo approach, agreed with independent ex vivo measurements of the dispersion difference between H_2_O and D_2_O (Supplementary Fig. S2f). Therefore, as the sample replaces the immersion medium during focus translation, the change in dispersion along the path to the focus can be reliably quantified and corrected.

### Volumetric imaging

**Fig. 2:**
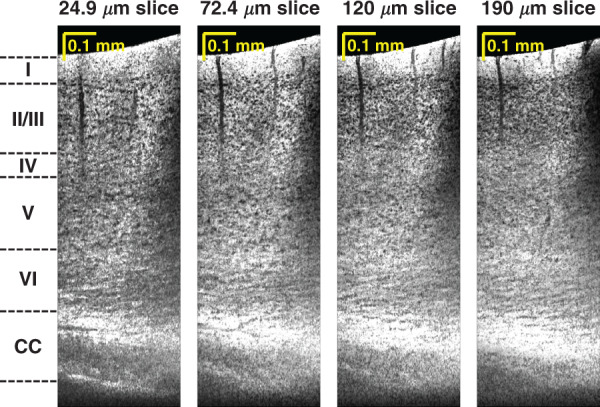
In vivo minimally invasive optical biopsy of the mouse brain. Minimum intensity sagittal images, with different projection thicknesses in the coronal direction, show cytoarchitecture across cortical depth, without requiring tissue slicing. Cortical layers and corpus callosum (CC) are labeled

**Fig. 3:**
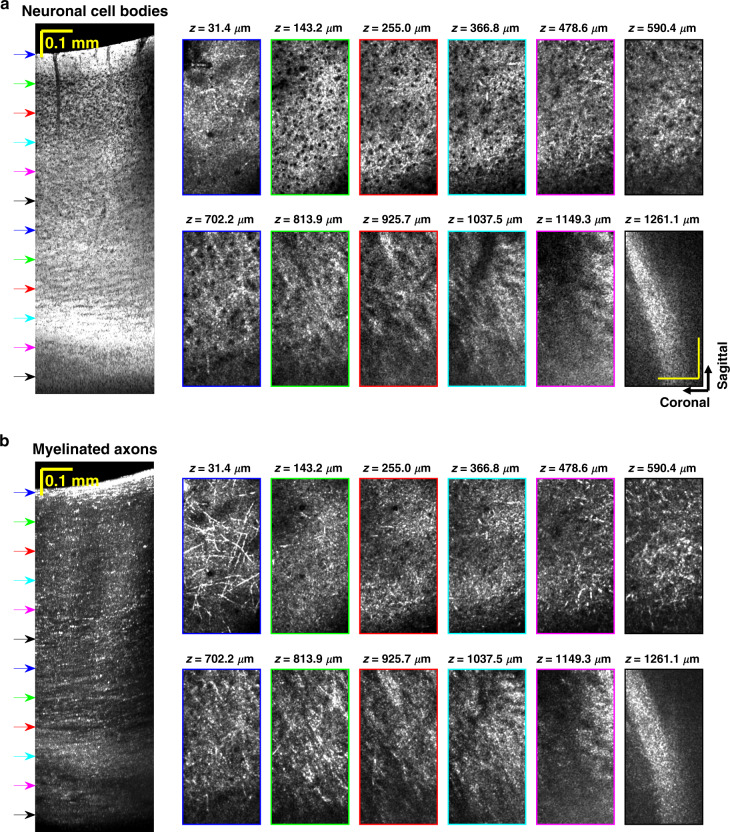
Transverse optical sectioning. In vivo visualization of neuronal cell bodies (**a**) and myelinated axons (**b**) in transverse planes. Outline colors of *en face* images correspond to arrow colors on the sagittal images on the left, indicating projection locations. Sagittal slice projection thickness: 190 μm. Axial projection depth: 11.2 μm. Scalebars represent 0.1 mm and apply to all the *en face* images

Sagittal minimum intensity projection images highlight neuronal cell bodies (Fig. 2) without cutting the brain. Laminar variations in the cell body distribution consistent with granular cortex^27^ are observable. Cytoarchitecture and myeloarchitecture trends are also visualized in transverse planes (Fig. 3). As might be expected, bulk tissue OCM attenuations (signal slopes) also differ across cortical layers (Supplementary Fig. S3), with neurite- and myelin-rich layer I showing a much larger attenuation coefficient than the layers underneath with higher cell density. While OCM signal slope is related to the ballistic attenuation coefficient, OCM signal slope is generally smaller in magnitude than ballistic attenuation due to detection of multiply scattered light^28^. OCM signal slope can also be affected by depth-dependent backscattering. For instance, the increase in backscattering due to increasing myelination with cortical depth across layer IV-VI^29^ likely contributes to a lower signal slope. A similar flattening of the three-photon excited fluorescence signal versus depth in deeper cortical layers was not observed^11,30^. These differences may arise from the physical mechanisms responsible for signal generation; fluorescence in three-photon microscopy and backscattering in OCM.

**Fig. 4:**
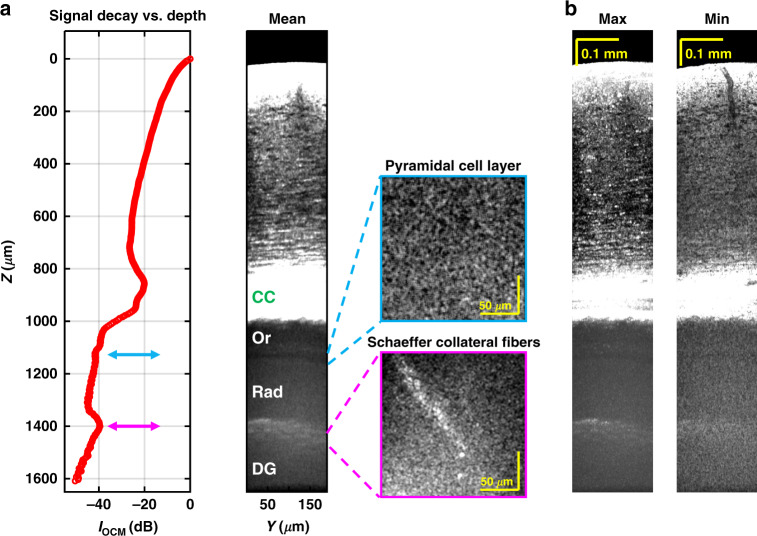
In vivo minimally invasive sub-cortical imaging. **a** OCM signal decay vs. depth (left panel) shows layered features in the sub-cortical region, as seen in the corresponding averaged coronal image (middle panel). CC: corpus callosum, Or: stratum oriens, Rad: stratum radiatum, DG: dentate gyrus. *En face* images of the Pyramidal cell layer and Schaeffer collateral fiber layer are shown in the right panel. *En face* image projection depth: 11.2 μm. **b** Maximum and minimum intensity projection coronal images, projected across a slab thickness of 60.0 μm

Overall, in agreement with an earlier Optical Coherence Tomography (OCT) study^17^, OCM signal in the 1700 nm optical window attenuates gradually with brain depth (Supplementary Fig. S3c), enabling imaging of sub-cortical regions (Fig. 4). Image volumes depict cellular detail in three dimensions (Supplementary Visualizations 1 and 2). Changes in the orientation and size of myelinated fibers from the mid-cortical to sub-cortical regions, more than 1 mm deep, are clearly visualized (Supplementary Fig. S4).

### Evidence for lipid absorption

**Fig. 5:**
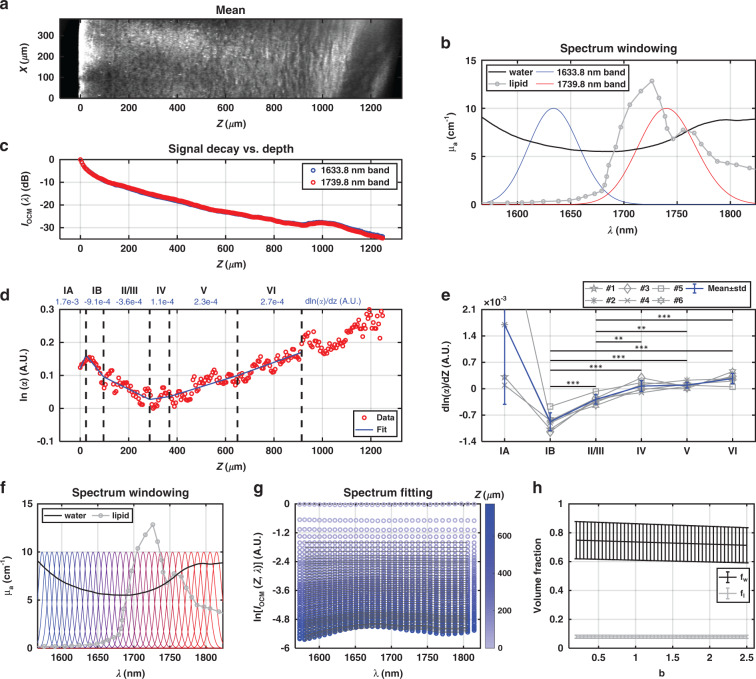
Spectroscopic tissue composition analysis. **a** Sagittal image, averaged over a 59.3 μm thick slab, showing morphological changes with depth (*Z*). Spectroscopic analysis was performed with either two wide subbands (**b**–**e**) or thirty-two narrow subbands (**f**–**h**). **b** Water absorption (black) is nearly identical between the two wide subbands, while lipid absorption (gray) is weighted towards longer wavelengths. **c** Background-corrected subband signal decay profiles exhibit slight differences. **d** The signal ratio between short and long wavelength subbands, analyzed according to layer. Natural logarithm of the signal ratio (*α*, red circles) versus depth is quantified using piecewise linear fitting (blue line) for each cortical layer. **e** Log signal ratio slopes of six animals (gray), with mean ± std. dev. (blue), reveals that signal ratio increases from layer IV to layer VI. **f** Subband windows for higher spectral resolution analysis. **g** Exemplary data from one animal showing OCM spectrum (logarithm scale) varies with depth (from shallower to deeper into cortex: light to dark blue, circles), and the corresponding fitting results (dark solid lines). Fitting ranges in *Z* and *λ* are indicated by the dark solid lines. **h** Mean ± std. dev. of water ($f_w$) and lipid ($f_l$) volume fraction estimates from six animals as the assumed b value (scattering power) changes

In addition to imaging of brain architecture (Fig. 5a), OCM potentially quantifies tissue composition through spectroscopic analysis of attenuation. Within the water absorption window^16^, lipid absorption^31^ is significantly higher beyond 1700 nm (Fig. 5b). The OCM signal at focal depth *Z* and wavelength *λ* is defined as:

1 $$I_{OCM}(Z,\lambda ) = \mu _b(Z,\lambda )I_0(\lambda )e^{ - 2{\int}_0^Z {\mu _t} (u,\lambda )du}$$

where $\mu _b$ represents the backscattering coefficient and $I_0$ is the reference OCM spectrum. The total tissue attenuation coefficient is defined as: $\mu _t\left( {Z,\lambda } \right) = \mu _{t,s}\left( {Z,\lambda } \right) + f_w(Z)\mu _{a,w}\left( \lambda \right) + f_l(Z)\mu _{a,l}\left( \lambda \right)$, where $\mu _{t,s}$ is the tissue scattering attenuation accounting for multiple scattering effects, $\mu _{a,w}\left( \lambda \right)$ and $\mu _{a,l}\left( \lambda \right)$ are water and lipid absorption coefficients, respectively. $f_w$ and $f_l$ represent water and lipid volume fractions. Due to the uncertainty principle inherent in the short time Fourier transform^32^, we performed two complementary analyses that trade depth resolution for spectral resolution. First, in a high depth resolution analysis, we investigated local lipid content changes with depth, employing the signal ratio (*α*) of the $\lambda _1 =$ 1633.8 nm subband to the $\lambda _2 =$1739.8 nm subband [subband window full-width-at-half-maximum (FWHM) in wavenumber: $1.3\, \times 10^5$ rad/m, Fig. 5c]:

2 $$\alpha = I_{OCM}(Z,\lambda _1)/I_{OCM}(Z,\lambda _2)$$

With the assumption that the backscattering ratio of the two subbands is invariant with depth, the derivative of the signal ratio can be approximated by (see Supplementary Section S4):

3 $$\frac{{d\ln (\alpha )}}{{dZ}} = 2[\mu _t(Z,\lambda _2) - \mu _t(Z,\lambda _1)]$$

Note that if scattering were the main source of attenuation, short wavelengths would be attenuated more, and *α* would decrease with depth. Higher absorption, and hence, attenuation, in the longer wavelength ($\lambda _2$) subband would increase *α* with depth. As seen in Fig. 5d, e, *α* clearly increases with depth in the deeper cortical layers. This suggests higher lipid absorption in the $\lambda _2$ subband.

Gaining confidence from this preliminary evidence of a lipid absorption signal, a second, high spectral resolution analysis was conducted. To estimate bulk volume fractions of cortical water and lipid, the OCM spectrum was analyzed with 32 subbands (subband window FWHM in wavenumber: $3.2\, \times 10^4$ rad/m, Fig. 5f) and fitted by the following model (Fig. 5g):

4 $$\ln \left[\frac{{I_{OCM}(Z,\lambda )}}{{I_0(\lambda )}}\right] = \mu _b(Z) - 2[\mu _{t,s}(\lambda ) + f_w\mu _{a,w}(\lambda ) + f_l\mu _{a,l}(\lambda )]Z$$

with the assumption that $\mu _b$ varies with *Z*. Scattering attenuation is assumed to be described as $\mu _{t,s}\left( \lambda \right) = A(\lambda /500)^{ - b}$, where *b* is the scattering power^33^. As *b* changes from 0.1 to 2.5, the mean recovered water volume fraction of six animals varies from 0.75 to 0.71, and the mean lipid volume fraction has a relatively consistent value of 0.08 (Fig. 5h), which are in agreement with the reported ex vivo measurements^22,34,35^.

### Neuropathology

**Fig. 6:**
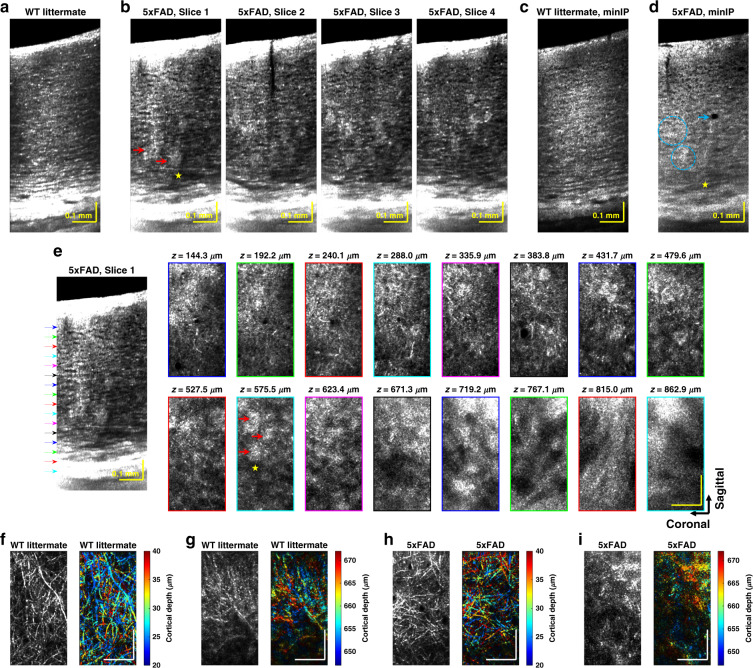
OCM of Alzheimer’s disease mouse model. Compared to the WT littermate (**a**), the 5xFAD transgenic mouse (**b**) has distinct highly scattering clusters (red arrows) and wide hyporeflective regions (yellow asterisk). Corresponding histology (Supplementary Fig. S7) suggests that the highly scattering clusters are plaques. Minimum intensity projection (minIP) image in the WT littermate (**c**) depicts clear cytoarchitecture, which is lacking in the AD mouse in deeper cortical layers (**d**, cyan circles). Hyporeflective voids, potentially related to tissue loss, are also noted (cyan arrow). **e** AD-associated plaques (red arrows) and regions of possible demyelination (yellow asterisk) are visualized in the *en face* plane. Outline colors of *en face* images correspond to arrow colors on the sagittal image on the left, indicating projection locations. Sagittal slice summation thickness: 17.8 μm. Depth color-coded *en face* images of myelinated axons and corresponding grayscale images illustrate intact myeloarchitecture in the WT littermate (**f**–**g**), while suggesting demyelination in deeper layers of the AD mouse (**h**–**i**). Taken together, OCM depicts a higher incidence of abnormal findings in layers IV-VI, consistent with the higher disease burden (Supplementary Fig. S7). Axial projection depth of 16.0 μm and scalebar size of 0.1 mm apply to all the *en face* images in **e**–**i**

To further demonstrate the utility of our approach for neuropathological studies in older mice, we proceeded to image a transgenic (5xFAD) model of Alzheimer’s disease. In this model, amyloid pathology in the cortex develops earliest in deeper layers^36,37^. Imaging the resulting variations with cortical depth presents a challenging test for any in vivo microscopic technique. Notably, the OCM approach does not require the common but artificial approaches of transgenic expression of a fluorescent protein or injection of a contrast agent^38^, greatly facilitating such studies. OCM images from a 14-month-old 5xFAD transgenic mouse and its wild type (WT) littermate are compared in Fig. 6 and Supplementary Figs. S5 and S6. Highly scattering clusters (indicated by red arrows) were observed in the AD volumes but not in WT volumes (Fig. 6a, b, and Supplementary Fig. S5), and corresponded with FSB-labeled amyloid plaques in histology (Supplementary Fig. S7a). Large hyposcattering regions in deeper cortical layers (example indicated by the yellow asterisk) were observed in AD volumes (Fig. 6b and Supplementary Fig. S7a). As NeuN was present in these regions (Supplementary Fig. S7a), we must invoke an explanation other than neuronal loss. One possible explanation for these hyposcattering regions is demyelination, which has been reported before^39,40^. Hyposcattering voids (one example indicated by the cyan arrow) are also seen in the AD minimum intensity projection (minIP) image (Fig. 6d). These voids, with lower reflectivity than surrounding neuropil, are spheroids with well-demarcated boundaries and present at shallower cortical depths in OCM. In minIP images, laminar cytoarchitectural trends are intact in the WT littermate (Fig. 6c), yet are disrupted in deeper layers of the AD mouse by plaques (Fig. 6d, cyan circles). Features of plaques, tissue loss, and degeneration, displayed in transverse planes of an AD mouse (Fig. 6e), are absent in the WT (Fig. 6f, g, and Supplementary Figs. S5 and S6). Notably, single myelin fibers are visualized in layer I of the 5xFAD mouse (Fig. 6h), as in the WT (Fig. 6f). However, relative to the WT (Fig. 6g), myelin contrast is lost in deeper cortical layers of the 5xFAD mouse where large plaques appear (Fig. 6i), consistent with above hypothesis of demyelination. The increasing disease burden with cortical depth^36^, as quantified by histological plaque count (Supplementary Fig. S7b), seems to be manifested in the increased incidence of abnormal OCM findings in layers IV-VI. Notably, minimal differences between the WT and 5xFAD mouse were observed in the superficial cortex, highlighting the importance of deep imaging in this model.

## Discussion

Neuroimaging techniques must strike a compromise between minimal invasiveness, resolution, imaging depth, and the use of exogenous contrast. The 1700 nm OCM approach introduced here achieves a unique balance between these competing goals. Here we explore two key aspects of the approach: the coherence gate and the long wavelength excitation.

### Benefits over confocal microscopy: the coherence gate

One advantage of OCM is the synergy between confocal and coherence gating to improve imaging depth. Confocal gating, achieved here by illumination and detection through a single mode optical fiber that acts as a pinhole^41^, provides depth sectioning in highly scattering tissue^42^. However, with deeper focusing, confocal performance degrades markedly due to multiple scattering. In this study, by filtering light according to path length^43^, the coherence gate enabled us to partially reject multiply scattered light.

Can we observe degradation of the confocal gate and quantitatively assess the benefits of the coherence gate? Referring to Supplementary Fig. S8a, b, the theoretical FWHM of the system confocal gate (red curve) is narrower than that of the coherence gate (blue curve). However, as the focusing depth increases, the range of light path lengths that pass the confocal gate broadens due to multiple scattering (Supplementary Fig. S8c, d). To answer the above question, we employ our multidimensional data set to digitally broaden the coherence gate, and thereby assess its benefits. As the effective, digitally broadened, coherence gate width ($\delta z_{eff}$) increases (Supplementary Fig. S8c, d), we find that the signal decays more slowly with focal depth (*Z*). It has previously been suggested that the signal decay with depth ($\mu _t$) should approach the ballistic attenuation coefficient, the sum of scattering and absorption coefficients ($\mu _s + \mu _a$), as rejection of multiply scattered light improves^28^, where a steeper signal slope indicates better rejection of multiply scattered light. Therefore, since a narrower coherence gate (i.e., finer OCM axial resolution) steepens the signal slope (Supplementary Fig. S8c), we infer that it aids selective removal of multiply scattered light. Thus, the use of the entire 1700 nm window bandwidth with an extended InGaAs detector, as opposed to only a portion of this window with standard InGaAs, aids deeper imaging.

### Longer wavelengths enable deeper OCM imaging

It is instructive to compare the performance of the 1700 nm window with the more popular 1300 nm window for deep OCT imaging. Previously 1300 nm OCM was shown to provide images of the deep cortical layers, but not subcortical regions, in rats, through invasive cranial windows^13^. The OCM signal slope at 1700 nm was estimated to be 35.3% lower than that at 1300 nm^17^. Approximating signal slope as a summation of terms corresponding to tissue scattering and absorption, and since water and lipid absorption are known to be higher at 1700 nm than at 1300 nm, we infer that attenuation due to scattering must be lower at 1700 nm. This points to a reduction in the scattering coefficient, though possible differences in scattering anisotropy cannot be ruled out^28,33^. Nonetheless, the minimally invasive, high quality and deep brain imaging achieved here by 1700 nm OCM provide empirical data to support the benefits of longer wavelength OCM.

In optical coherence tomography (OCT) and OCM, moving to longer wavelength has been considered as a dual-edged sword, since, though attenuation is reduced, backscattering may be reduced too. The observed performance of longer wavelengths for deep imaging in this work comports with results of brain tissue clearing studies^29,44^, where the benefits of a reduction in multiply scattered light far outweigh the reduction in backscattering cross-section, aiding deeper imaging. Nevertheless, the ability to resolve features in vivo does degrade slightly across the cortical depth (Supplementary Fig. S9). While the technical challenges of the 1700 nm wavelength range are significant, improvements in light sources and detector technology are on the horizon. The demonstrated capabilities of 1700 nm OCM for imaging deep in the brain suggest promise for deep imaging in other highly scattering, water-rich tissues as well.

## Materials and methods

### System design and characterization

**Fig. 7:**
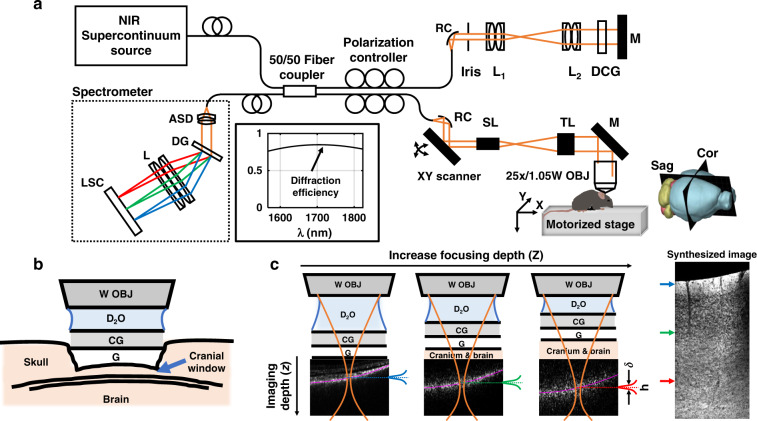
System design and experimental setup. **a** Schematic of the 1700 nm OCM system. RC: reflective collimator, L_1_, L_2_: lenses (achromatic doublet pairs) in the reference arm, DCG: dispersion compensation glass, M: mirror, SL: scan lens, TL: tube lens, 25x/1.05 W OBJ: 25x NA 1.05 water immersion objective, Sag/Cor: sagittal/coronal plane, ASD: air-spaced doublet, DG: diffraction grating, L: lens, LSC: line scan camera. The 3D mouse brain rendering was obtained from the Brain Explorer 2 software (http://mouse.brain-map.org/static/brainexplorer). Inset (black box) shows the diffraction efficiency of the customized grating over the system spectral band. **b** Preparation for thinned-skull imaging. W OBJ: water immersion objective, D_2_O: heavy water immersion medium, CG: cover glass, G: glue. **c** Dynamic focusing and image synthesis. The sample is translated stepwise towards the objective. As the focus translates deeper into the brain, the cranium and brain replace the immersion medium in the light path. The axial weighting function *h*, with width *δ*, multiplies each volume prior to image fusion and display

**Fig. 8:**
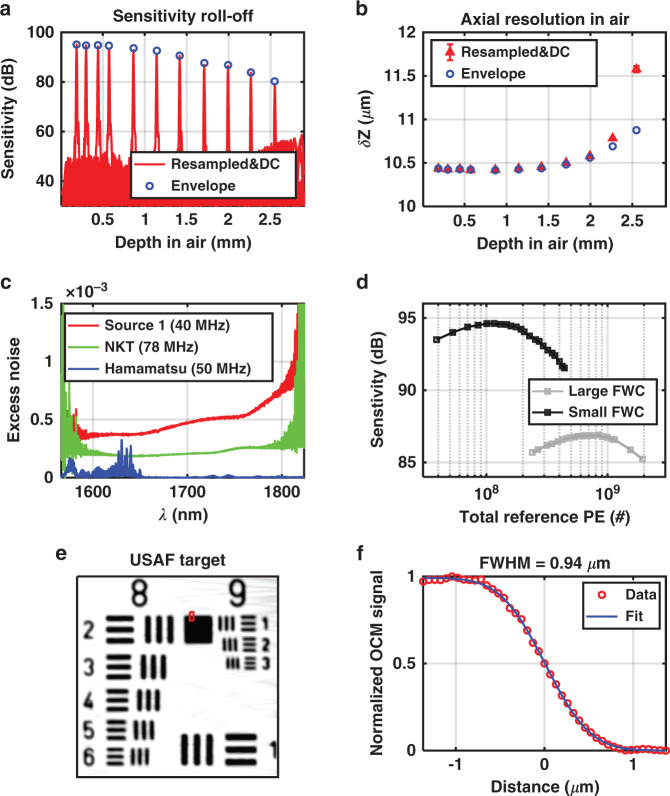
1700 nm OCM system characterization. **a** Sensitivity roll-off and **b** axial resolution versus depth. To assess efficacy of resampling and dispersion compensation (DC), results (red) are compared with theoretical values from the envelope (blue circles). **c** Excess noise coefficient measurements of three light sources. **d** System sensitivity using Hamamatsu light source with large (12.5 Me^−^) or small (1.25 Me^−^) full well capacity (FWC) camera settings. **e** *En face* image of groups 8 and 9 of a negative USAF 1951 target. **f** Measured edge response (red circles), from the red box in **e**, are fitted with an error function (blue line) to yield the transverse resolution

As shown in Fig. 7a, light from a near-infrared (NIR) supercontinuum light source (L15077-C7-Y001, Hamamatsu, Corp., NJ, USA) is split into reference and sample arms by a 50/50 SMF-28 customized fiber coupler (Haphit, Inc., Shanghai, China). In both arms, the beam is collimated by a 15 mm effective focal length (EFL) reflective collimator (RC04APC-P01, Thorlabs, Inc., NJ, USA). In the sample arm, the beam is scanned by a 2D galvanometer (6215H, Cambridge Technology, Inc., MA, USA), magnified by a scan lens and tube lens pair (SL50-3P & TL200-3P, Thorlabs, Inc.), and then focused onto the sample using a high numerical aperture (NA) water immersion objective (XLPLN25XWMP2, Olympus America, Inc., PA, USA). Sample translation is controlled by motorized stages in the transverse ($X,Y$, ILS50CC, Newport, Corp., CA, USA) and axial (*Z*, L-310, Physik Instrumente, L.P., MA, USA) directions. In the reference arm, the beam is magnified by two pairs of achromatic doublets (Thorlabs, Inc.), with 50 mm and 75 mm EFL. A glass block approximately matches dispersion in the sample arm and an adjustable iris controls reference power. The backscattered light from the sample and light reflected from the reference mirror are then combined by the fiber coupler and delivered to the spectrometer. In the spectrometer, light is collimated by an air-spaced achromatic doublet (ACA254-075-D, Thorlabs, Inc.), dispersed by a custom 1700 nm diffraction grating (600 lines per millimeter, Wasatch Photonics, Inc., UT, USA), focused by an achromatic doublet (150 mm EFL, Thorlabs, Inc.), and detected by an extended InGaAs line scan camera (SU1024LDH-2.2RT, Sensors Unlimited, Inc., NJ, USA). Data were collected via the frame grabber (PCIE-1427, National Instruments, Corp., TX, USA). Sensitivity roll-off and axial resolution degradation are insignificant over the first millimeter depth, which suffices for high NA, short depth-of-field, imaging (Fig. 8a, b). The camera and 2D galvanometer scanner were synchronized using a custom LabVIEW program. As described in the subsequent sections, the system design was tailored to maximize sensitivity through reducing source excess noise and improving spectrometer efficiency, while minimizing aberrations and dispersion.

#### Reduced excess noise

To take full advantage of the entire 1700 nm water absorption optical window, which extends beyond the cutoff of standard InGaAs, an extended InGaAs camera was employed for detection. However, due to lattice mismatch, extended InGaAs exhibits higher detector noise than standard InGaAs. The elevated detector noise, in conjunction with excess light source noise, compromises the ability to achieve shot noise limited (SNL) sensitivity^45^. To maximize the system sensitivity, three commercial prototype supercontinuum light sources (#1: Source 1; #2: SuperK Extreme, NKT Photonics, Inc., MA, USA; #3: L15077-C7-Y001, Hamamatsu Corp.) were characterized and compared (Fig. 8c). The first two were photonic crystal fiber (PCF) based broadband supercontinuum sources, while the third was a Hamamatsu prototype, in which a pulse centered at 1690 nm was filtered from multiple soliton orders after fission in a single mode fiber, followed by further spectral broadening by self-phase modulation in a normal dispersion highly nonlinear fiber^46^. The repetition rates of the two PCF-based supercontinuum sources were 40 and 78 MHz, while the repetition rate of the Hamamatsu source was 50 MHz. The Hamamatsu source was found to provide the highest system detection sensitivity (94.6 dB with 3.5 mW sample power and an exposure time of 14.1 μs), and was chosen for this study (Fig. 8d).

#### Focal shifting

A tightly focused spot favors cellular resolution in the *en face* plane. In the sample arm, an optimized scan and tube lens pair is used, which achieves an Airy disk confined spot diagram based on the ray tracing simulation. The experimentally measured transverse resolution of 0.94 μm is near the theoretically calculated value of 0.77 μm (Fig. 8e, f). The corresponding confocal-gated depth-of-field of 3.2 μm (intensity FWHM in tissue), together with the coherence gate of 5.6 μm (intensity FWHM in tissue), defines the axial resolution. To perform volumetric imaging, the sample is translated stepwise towards the objective, in 5 μm intervals (Fig. 7c). Relative to confocal microscopy, we improve sectioning by employing the path length resolution achieved by the coherence gate to filter ballistic or quasi-ballistic signal from the purported focus (Fig. 7c).

### Surgical preparation

Six adult wild type mice (four 2–4-month-old C57BL/6 males, as well as 3.3-month-old and 12.5-month-old C57BL/6 females, from Charles River), one five-familial Alzheimer’s disease (5xFAD) transgenic mouse, and its WT littermate (both 14-month-old males) were imaged. Mice were anesthetized continuously with isoflurane (1–2.5% v/v), vaporized in a gas mixture of 80% medical air and 20% oxygen. Mice were immobilized in a stereotactic frame (Stoelting Co., IL, USA), while head fixed by ear pins and a bite bar. As shown in Fig. 7b, the scalp was then removed carefully, and a thinned-skull cranial window centered near the posterior somatosensory cortex was created with a dental drill under saline cooling. The skull was not thinned aggressively, leaving a residual thickness of 49.3 ± 11.6 μm (mean ± std. dev.) to minimize possible mechanical trauma to the superficial cortex. Next, a coverslip was attached to the skull with superglue (Vibra-Tite, FL, USA). D_2_O, with a lower absorption than H_2_O, was added as the objective immersion medium.

For an imaging session that acquired at foci from the cortical surface ($Z = 0$ mm) to a cortical depth of $Z = 1.5$ mm, 301 volumes were acquired with a translation step $D_Z$ of 5 $\mu m$. A total of 22 min was required for stage translation, scanning, and data saving [1024 (*z*) x 640 (*X*) x 320 (*Y*) voxels per volume]. This constitutes a four-dimensional (*z*, *X*, *Y*, *Z*) data set. The incident sample power was 3.5 mW. Note that to achieve similar imaging depths, state-of-the-art nonlinear techniques such as multi-photon microscopy^12,47^ or third-harmonic-generation microscopy^48^ require an order-of-magnitude higher power, even with more invasive preparations. All the experimental procedures and setup were approved by UC Davis Institutional Animal Care and Use Committee.

### Dynamic focusing and image fusion

OCM image reconstruction consisted of background subtraction, fringe resampling, spectral shaping, dispersion compensation, and Fourier transformation. Volumetric complex OCM data at each focusing depth *Z*_*i*_, indexed by *i*, are denoted as $A_i(z,X,Y)$, where *z*, *X*, *Y* are OCM coordinates. Note that *z*, the OCM depth, is determined as the path length divided by 2, divided by an assumed refractive index of 1.33. The synthesized image volume $I(z,X,Y)$ is given by (Fig. 7c):

5 $$I(z,X,Y) = \mathop {\sum}\limits_{i = 1}^N {|A_i(z - S_i,X,Y)|^2} h\left[ {z - F_i(X,Y) - S_i} \right]$$

where $S_i = (i - 1)D_S$ represents the depth shift of the volumetric data, with depth shift $D_S$ being related to the sample translation interval $D_Z$ (they are equal if the group index of the immersion medium is assumed for OCM reconstruction). The axial weighting function, *h*, suppresses out-of-focus and multiply scattered light. It was chosen as the convolution (*) of rectangular and Gaussian functions, $h\left( z \right) = Rect\left[ {z/(10.6\;\mu m)} \right] \ast exp\left[ { - z^2/(14.2\;\mu m^2)} \right]$. The FWHM of *h* (${\updelta} = 11.4\mu m$) was carefully optimized to both suppress unwanted light, while also reducing speckle (Supplementary Fig. S10). $F_i(X,Y)$ denotes the estimated focus along the depth (*z*) axis (see Supplementary Section S10). The approximate physical focusing depth is denoted by *Z*_*i*_ or *Z* in plots. Sub-pixel shifting in the axial direction was performed on the complex data *A*_*i*_ via the Fourier shift theorem. Image reconstruction and fusion in post processing took 15.4 h in Matlab (R2020a, MathWorks, Inc., MA, USA) on a workstation with the Xeon W-2135 Processor @3.7 GHz (Intel, Corp., CA, USA). This time can be improved by optimizing the code for parallel processing.

### Display

Before display, the synthesized image volume was first normalized in depth (*z*) to account for tissue attenuation. Then 2D averaging (3.0 μm) in *X* and *Y*, together with image fusion in *z*, generates the 3D averaged image volume.

#### Cytoarchitecture

Hypo-reflective neuronal cell bodies are distinguished from the surrounding neuropil by lower backscattering^13^. Thus, minimum intensity projections were taken across slabs within the image volume for in vivo visualization of cytoarchitecture in sagittal, coronal, or transverse planes.

#### Myeloarchitecture

Axons with a high refractive index, lipid-rich myelin sheath are distinguished from the surrounding neuropil by higher backscattering^13,49^. Thus, maximum intensity projections were taken across slabs within the image volume for in vivo visualization of myeloarchitecture in sagittal, coronal, or transverse planes.

#### Plaques

Insoluble Aβ aggregates or plaques are highly scattering compared to background neuropil in the mouse cerebral cortex^50^. To visualize highly scattering clusters corresponding to plaques, 3D imaging volumes were summed over the imaging projection direction.

### Fitting laminar signal characteristics

In addition to visualizing cytoarchitecture and myeloarchitecture, laminar variations in optical properties may relate to tissue composition. Layer-by-layer tissue attenuation coefficients, taken as the change with focal depth (i.e., slope) of the OCM signal around the estimated focus, were quantified by piecewise linear fitting. Spectroscopic analysis of attenuation was performed, and interpreted in terms of water and lipid content.

## Supplementary information

Supplementary Information

1700 nm cellular deep brain optical biopsy, Visualization 1

1700 nm cellular deep brain optical biopsy, Visualization 2

## Data Availability

All data needed to evaluate the conclusions in the paper are present in the paper and/or the Supplementary Materials. Additional data related to this paper may be available from corresponding author upon reasonable request.
