# Supplementary material for: 1700 nm optical coherence microscopy enables minimally invasive, label-free, in vivo optical biopsy deep in the mouse brain
Source: Light Sci Appl. 2021 Jul 14;10:145. doi: 10.1038/s41377-021-00586-7 (PMC8280201; doi:10.1038/s41377-021-00586-7)
Supplement: Supplementary file 1 — Supplementary Information [file 41377_2021_586_MOESM1_ESM.pdf]

Supplementary Information for

1700 nm optical coherence microscopy enables minimally invasive, label-free, *in vivo* optical biopsy deep in the mouse brain

Short title: 1700 nm cellular deep brain optical biopsy

Jun Zhu[1], Hercules Rezende Freitas[2], Izumi Maezawa[2], Lee-way Jin[2], and Vivek J. Srinivasan[1,3,4,5,6,*]

[1]Department of Biomedical Engineering, University of California Davis, Davis, California, 95616, USA

[2]Department of Pathology and Laboratory Medicine, University of California Davis Medical Center, Sacramento, California, 95817, USA

[3]Department of Ophthalmology and Vision Science, School of Medicine, University of California Davis, Sacramento, California, 95817, USA

[4]Department of Ophthalmology, NYU Langone Health, New York, New York, 10017, USA

[5]Department of Radiology, NYU Langone Health, New York, New York, 10016, USA

[6]Tech4Health Institute, NYU Langone Health, New York, New York, 10010, USA

Jun Zhu: znjzhu@ucdavis.edu

Hercules Rezende Freitas: herculesrezendef@hotmail.com

Izumi Maezawa: imaezawa@ucdavis.edu

Lee-way Jin: lwjin@ucdavis.edu

*Vivek J. Srinivasan: vjsriniv@ucdavis.edu, Phone: 530-752-9277

Abstract

This document provides supplementary information for “1700 nm optical coherence microscopy enables minimally invasive, label-free, *in vivo* optical biopsy deep in the mouse brain”. Here, we provide a more complete description of the optical coherence microscopy (OCM) system. We propose a robust, simple and novel approach for OCM chromatic dispersion quantification and numerical compensation, which is validated by independent experimental measurements. We show features of cortical lamination and myeloarchitecture from the mid-cortical to sub-cortical regions. Spectroscopic OCM estimation of cortical tissue composition is described. 3D visualizations of the 5xFAD Alzheimer’s disease transgenic mouse and its wild type littermate are provided. A co-registered comparison of OCM and histology is presented. In addition, the benefits of OCM over confocal microscopy are quantified. Degradation in the ability to resolve features with depth *in vivo* is estimated. We also illustrate optimization of the weighting function used for image fusion and display. Finally, our method of detecting the OCM depth of the focus is presented.

S1. Chromatic dispersion compensation

As the optical coherence microscopy (OCM) spectrum spanned a broad spectral range from 1560 to 1820 nm (**Fig. S1**a), chromatic dispersion must be addressed to optimize axial image resolution. Dispersion mismatch between the sample and reference arms induces a nonlinearity in the spectral phase, so the point spread function (PSF) is chirped and broadened (**Fig. S1**b-c). Numerical dispersion compensation in post-processing can remove this spectral phase and optimize the OCM image quality. Unfortunately, reliable published data for dispersion of heavy water (D_2O) and water (H_2O) in this wavelength range are lacking, and it is uncertain if dispersion changes during focus translation as D_2O is replaced by brain tissue along the optical path. Image-based metrics to optimize numerical dispersion compensation include image sharpness[1], local image contrast[2] and alignment of subband images[3]. However such approaches are challenging to implement in images that lack well-defined features.

We empirically observed that optimal dispersion compensation minimized the width of the distribution of path lengths around the nominal focus, which we called the "apparent focal width (AFW)". Remarkably, this observation held even when this distribution was significantly broadened due to light scattering while focusing deep into the sample (**Fig. S1**d). Based on this observation, we chose to optimize numerical dispersion compensation by minimizing the apparent focal width. The nonlinear spectral phase compensation ($\emptyset_{NL}$) is:

$$\phi_{NL} = \frac{1}{2} d_2 (\omega - \omega_0)^2 + \frac{1}{6} d_3 (\omega - \omega_0)^3, \tag{S1}$$

where ω is the angular optical frequency, ω_0 represents the central optical frequency, and d_2 and d_3 are coefficients compensating the group delay dispersion and third-order dispersion mismatches between the sample and reference arms, respectively. For given values of d_2 and d_3, numerical calculation of the apparent focal width is:

$$AFW(d_2, d_3) = \frac{1}{M} \sum_{j=1}^{M} W_j, \tag{S2}$$

where W_j is the full width at X_j% of the maximum PSF value. For this work, X_j values from 60% to 85% with 1% interval were used; therefore, $M = 26$. For each depth, width broadening (WB) is defined as:

$$WB = AFW(d_2, d_3) - AFW_0, \quad \text{(S3)}$$

where minimum apparent focal width (AFW_0) is subtracted from all AFW values. Surface plots (grid search) indicate changes of AFW and WB with coefficients d_2 and d_3 at different focal depths (**Fig. S1**d-e). Final optimized d_2 and d_3 values obtained by fminsearch[4] are consistent with grid search results. The fminsearch finds parameters to minimize AFW iteratively, requiring around 30 cycles to converge. As shown in **Fig. S1**f, optimum second-order dispersion compensation value increases by ~507 fs^2, while systematic changes in the third-order value are undetectable, as focal depth increases from 0 to 900 μm. This suggests that second-order dispersion dominates PSF broadening as brain tissue replaces D_2O along the optical path. This empirical result is directly confirmed in the next section (**Fig. S2**).

S2. Dispersion measurements of H_2O and D_2O over the entire 1700 nm optical window

Besides empirically assessing the dispersion *in vivo* in brain tissue via the AFW in the previous section, we also used the 1700 nm OCM system to measure the dispersion generated as H_2O directly replaces D_2O in a cuvette. While this approach enabled a direct assessment of dispersion differences between H_2O and D_2O, without the complication of scattering tissue, it is important to keep in mind that brain tissue is only ~75% water and its chromatic dispersion may differ from pure H_2O.

Briefly, a 2 mm cuvette was inserted into the reference arm for dispersion measurements[5]. The spectral phase of the OCM interferogram was determined (**Fig. S2**a) when the cuvette was empty (filled with air, top panel) or filled with either H_2O or D_2O (bottom panel). The spectral phases were subtracted to yield:

$$\Phi = 2\left[k_{medium}(\omega) - k_{air}(\omega)\right]L + \Phi_{res.}, \quad \text{(S4)}$$

where Φ is the spectral phase change between the medium (H_2O or D_2O) and air, k_{medium} and k_{air} are medium and air wavenumbers, respectively, ω is optical angular frequency, L is the cuvette length (2 mm), and $\Phi_{res.}$ is an unknown residual phase drift. As shown in **Fig. S2**b-c, the spectral phase change caused

by replacing 2 mm (4 mm double pass) air with H_2O (black solid line) versus D_2O (black dashed line) are not the same. The nonlinear part of the spectral difference ($\Delta\Phi_{NL}$, blue curve), representing the difference between H_2O and D_2O, is essentially what causes PSF broadening (**Fig. S2**c). With this measured nonlinear phase, PSF broadening was predicted as the OCM focusing depth increases from 0 to 2 mm (4 mm double pass) deep in water (**Fig. S2**d-e). Second-order or group delay dispersion (GDD) was shown to be dominant (**Fig. S2**e). Importantly, the *ex vivo* measurements of dispersion when H_2O replacing D_2O agree with results of optimized *in vivo* dispersion correction (**Fig. S2**f). Also, as suggested by the larger optical phase change across the spectrum, the group refractive index ($c \times \partial k_{medium}/\partial \omega$, where c is the speed of light) of H_2O was found to be 1.012 to 1.022 times that of D_2O across the spectrum (**Fig. S2**g-i).

S3. *In vivo* biopsy: cortical lamination and myeloarchitecture pattern

OCM visualizes laminar cytoarchitecture and myeloarchitecture *in vivo* (**Fig. S3**a) and quantifies the signal attenuation across the cortex (**Fig. S3**b-c). We also show variations of myeloarchitecture pattern from mid-cortical to sub-cortical regions using OCM *in vivo* biopsy. As shown in **Fig. S4**, many short, oblique axons present in mid cortex ($Z < 650\,\mu m$), therefore, they appear as individuals with different orientations in transverse planes; whereas in deeper cortical layer ($650\,\mu m < Z < 900\,\mu m$), axons orient in the antero-posterior direction, with few exceptions, therefore, they show up as parallel groups perpendicular to the coronal plane[6]. In corpus callosum and deeper regions ($Z > 900\,\mu m$), large fiber bundles form and their orientations vary with depth.

S4. Estimation of cortical composition

Here, we show the derivation of subband OCM signal ratio for local lipid component change estimation. Starting from Eq. (1), the OCM signal at focal depth Z and wavelength λ is given by:

$$I_{OCM}(Z,\lambda) = \mu_b(Z,\lambda) I_0(\lambda) e^{-2\int_0^Z \mu_t(u,\lambda)du}, \quad \text{(S5)}$$

where μ_b represents the backscattering coefficient, I_0 is the reference OCM signal which is typically set at the cortical surface, and μ_t is the total attenuation coefficient. The signal ratio of two subbands is:

$$\alpha = \frac{I_{OCM}(Z,\lambda_1)}{I_{OCM}(Z,\lambda_2)} = \frac{I_0(\lambda_1)\mu_b(Z,\lambda_1)}{I_0(\lambda_2)\mu_b(Z,\lambda_2)} e^{2\int_0^Z [\mu_t(u,\lambda_2)-\mu_t(u,\lambda_1)]du}. \quad \text{(S6)}$$

The natural logarithm of the signal ratio is:

$$\ln(\alpha) = C_1 + \ln[\frac{\mu_b(Z,\lambda_1)}{\mu_b(Z,\lambda_2)}] + 2\int_0^Z [\mu_t(u,\lambda_2) - \mu_t(u,\lambda_1)]du, \quad \text{(S7)}$$

where C_1 is the reference subband ratio constant. Assuming backscattering ratio of two subbands is fixed with depth, the derivative of $\ln(\alpha)$ with respect to Z becomes:

$$\frac{d\ln(\alpha)}{dZ} = 2[\mu_t(Z,\lambda_2) - \mu_t(Z,\lambda_1)]. \quad \text{(S8)}$$

We recall that $\mu_t(Z,\lambda)$ consists of scattering attenuation [$\mu_{t,s}(Z,\lambda)$], water absorption [$f_w(Z)\mu_{a,w}(\lambda)$], and lipid absorption [$f_l(Z)\mu_{a,l}(\lambda)$]:

$$\mu_t(Z,\lambda) = \mu_{t,s}(Z,\lambda) + f_w(Z)\mu_{a,w}(\lambda) + f_l(Z)\mu_{a,l}(\lambda), \quad \text{(S9)}$$

where f_w and f_l represent water and lipid volume fraction, and $\mu_{a,w}$ and $\mu_{a,l}$ are water and lipid absorption coefficient, respectively. In summary, the total attenuation difference between the two subbands determines slope of $\ln(\alpha)$, which can help infer changes in tissue components with cortical depth.

S5. OCM imaging of wild type littermate versus AD mouse

In contrast to the five-familial Alzheimer's disease (5xFAD) transgenic mouse, its wild type (WT) littermate does not present features such as plaques, tissue loss and myelin degeneration (**Fig. S5**). Myelinated axons are clearly visible in the WT littermate (**Fig. S6**a), while appearing diminished in deeper layers of the AD mouse (**Fig. S6**b).

S6. Comparison of *in vivo* OCM imaging with *ex vivo* histology

A comparison between *in vivo* OCM and the corresponding anti-NeuN and FSB-stained histology was performed in the AD mouse. Briefly, after OCM imaging, the mouse was immediately sacrificed, and the brain was excised and fixed with 10% formalin. Then the fixed sample was embedded in Paraffin and sliced at 4 microns for imaging. Histology slices were co-stained with anti-NeuN (Abcam, MA, USA) and FSB (Sigma-Aldrich, MO, USA), and imaged with a commercial microscope (Nikon, NY, USA) at 10x magnification. Exposure time and gamma were adjusted to optimally visualize NeuN and FSB in individual images, which were combined as red and blue channels, respectively, of a single color image. Anatomical features depicted by the two modalities correspond (**Fig. S7**a). A hyporeflective shadow in OCM corresponds with a blood vessel (cyan arrow). Hyperscattering clusters in OCM correspond with FSB-labelled plaques (green arrows), though smaller FSB-labelled plaques are not always visualized on OCM. NeuN is seen in regions corresponding to hyporeflective regions in OCM (yellow asterisks), therefore low scattering in OCM is proposed to be related with demyelination, rather than neuronal loss. Plaque density was estimated both from the OCM volume and from histology (**Fig. S7**b). OCM appears to underestimate the plaque density relative to histology, but does correctly depict the trend of increasing plaque load with cortical depth (**Fig. S7**b). Differences between plaque densities estimated by OCM and histology could be due to the imaging contrast[7,8]. In OCM, the ability to detect plaques is affected by local contrast between the plaque backscattering, determined by composition and morphology, and the surrounding tissue backscattering background. Therefore, it is possible that our OCM is detecting a subpopulation of the amyloid plaques highlighted by FSB.

S7. Benefits over confocal

Compared to confocal microscopy[9], the OCM approach better rejects multiply scattered and out-of-focus light. Coherence gating is achieved by a broadband light source (**Fig. S8**a, blue, δz) and confocal gating is achieved by a high numerical aperture (NA) water immersion objective (**Fig. S8**a, red, $2z_0$). Intensity profiles of the two gating effects in tissue show that confocal gating has a narrower full-width-at-half-

maximum (FWHM). However, we notice that the asymptotic decay of the confocal gate is more gradual than that of the coherence gate (**Fig. S8**b), suggesting that the coherence gate can further enhance the confocal gate.

The OCM approach provides a path length filter to selectively remove out-of-focus and multiply scattered light. Here, we demonstrate this concept by investigating the OCM signal slope as a function of the effective coherence gate width (δz_{eff}). At each focus location (Z), OCM intensity signal is 3D summed with different axial ranges, where δz_{eff} is the width of the coherence intensity profile convolved with a rectangular function that delineates the axial (depth) summation range. As δz_{eff} increases, OCM signal decays slower with depth (**Fig. S8**c-d), indicating increased detection of multiply scattered light[10]. This suggests that an OCM system that achieves high axial resolution by utilizing the entire water absorption window at 1700 nm rejects multiply scattered light more effectively than a system that only partially utilizes the 1700 nm window.

S8. *In vivo* characterization of resolution

While the system resolution was characterized *in vitro* in the main manuscript (**Fig. 8**), resolution may degrade *in vivo* due to multiple scattering and aberrations. To assess lateral (transverse) resolution degradation *in vivo*, we relied on salient OCM features: cell bodies and myelinated axons. In the axial direction, we used broadening of the apparent focal width (AFW) as an indirect indicator of broadening due to multiple scattering, which is the main source of degradation of both the PSF and AFW when imaging deep, if dispersion is compensated (**Fig. S1**). For each focus location, AFW was estimated (**Fig. S9**a-b). AFW increases with a deeper focus (**Fig. S9**c). In the transverse direction, line profiles of neuronal cell body edges and myelin were used to indicate the lateral resolution. Neuronal cell bodies were emphasized by minimum intensity projection (**Fig. S9**d), while myelinated axons were emphasized by maximum intensity projection (**Fig. S9**g). Regions of interests (ROIs) from the cell body edges were selected (as shown in **Fig. S9**d) and averaged perpendicular to the cell body edge to generate the edge or step

response. Then, the data was fitted with an error function to indirectly yield the lateral FWHM of the point or impulse response (**Fig. S9**e). For myelin, FWHMs were extracted directly from line profiles perpendicular to the axon axis (**Fig. S9**g-h). For both the cell body and myelin, a slight increase with depth is observed (**Fig. S9**f, i), suggesting resolution degradation *in vivo*. Note that this analysis provides evidence of resolution degradation, not direct estimates of resolution, since minimum or maximum intensity projections were analyzed, and since the intrinsic widths of the myelinated axon and cell body edge were neglected.

S9. Despeckle vs. out-of-focus light rejection

By combining coherence and confocal gates, optical coherence microscopy rejects multiply scattered and out-of-focus light. Image fusion in depth (z) is intended to average structures in adjacent data volumes to reduce speckle. However, a structure that is in focus in one volume is slightly out-of-focus in the next. Therefore, a weighting function, h, which balances speckle reduction against out-of-focus light suppression (**Fig. S10**a-b), multiplied each data volume prior to image fusion. The weighting function is determined as the convolution ($*$) of rectangular and Gaussian functions:

$$h(z) = rect[z/(2z_w)] * e^{-2z^2/z_w^2}, \qquad \text{(S10)}$$

where z_w adjusts the width of h. As shown in **Fig. S10**c, when h gets narrower, the contrast of myelinated axons against the background neuropil is enhanced due to better rejection of multiply scattered light, however, less averaging leads to an image that is more corrupted by speckle. A weighting function FHWM (δ) of 11.4 μm was chosen to balance the two effects.

S10. Focus detection

Here we describe our procedure to find the OCM depth (z) of the focus [$F_i(X,Y)$] for physical focusing depth Z_i, prior to weighting and image fusion. First, the maximum intensity location at each (X,Y) position is taken as a coarse approximation of the focus. This first estimate is noisy due to speckle. Next, a two-dimensional surface fit [up to second order with (X,Y) as variables] generates the smoother curve. Next,

the OCM focus depths at each (X, Y) coordinate are fitted by piecewise linear fitting versus physical depth Z. The slope in layer I is presumed to be different from that of other layers. This fit or interpolation also corrects for focus detection errors caused by anatomical features (for instance, highly scattering white matter biases the OCM depth of the focus inferred from the maximum intensity alone).

References

1 Wojtkowski, M. *et al.* Ultrahigh-resolution, high-speed, Fourier domain optical coherence tomography and methods for dispersion compensation. *Optics express* **12**, 2404-2422 (2004).

2 Xu, D., Huang, Y. & Kang, J. U. Compressive sensing with dispersion compensation on non-linear wavenumber sampled spectral domain optical coherence tomography. *Biomedical Optics Express* **4**, 1519-1532 (2013).

3 Kho, A. & Srinivasan, V. J. Compensating spatially dependent dispersion in visible light OCT. *Optics letters* **44**, 775-778 (2019).

4 Lagarias, J. C., Reeds, J. A., Wright, M. H. & Wright, P. E. Convergence properties of the Nelder--Mead simplex method in low dimensions. *SIAM Journal on optimization* **9**, 112-147 (1998).

5 Zhang, T., Kho, A. M. & Srinivasan, V. J. Water wavenumber calibration for visible light optical coherence tomography. *Journal of Biomedical Optics* **25**, 090501 (2020).

6 Morrison, J. H., Grzanna, R., Molliver, M. E. & Coyle, J. T. The distribution and orientation of noradrenergic fibers in neocortex of the rat: an immunofluorescence study. *Journal of Comparative Neurology* **181**, 17-39 (1978).

7 Gesperger, J. *et al.* Comparison of intensity-and polarization-based contrast in amyloid-beta plaques as observed by optical coherence tomography. *Applied Sciences* **9**, 2100 (2019).

8 Lichtenegger, A. *et al.* Ex-vivo Alzheimer's disease brain tissue investigation: a multiscale approach using 1060-nm swept source optical coherence tomography for a direct correlation to histology. *Neurophotonics* **7**, 035004 (2020).

9 Xia, F. *et al.* In vivo label-free confocal imaging of the deep mouse brain with long-wavelength illumination. *Biomedical optics express* **9**, 6545-6555 (2018).

10 Jacques, S. L., Wang, B. & Samatham, R. Reflectance confocal microscopy of optical phantoms. *Biomedical optics express* **3**, 1162-1172 (2012).

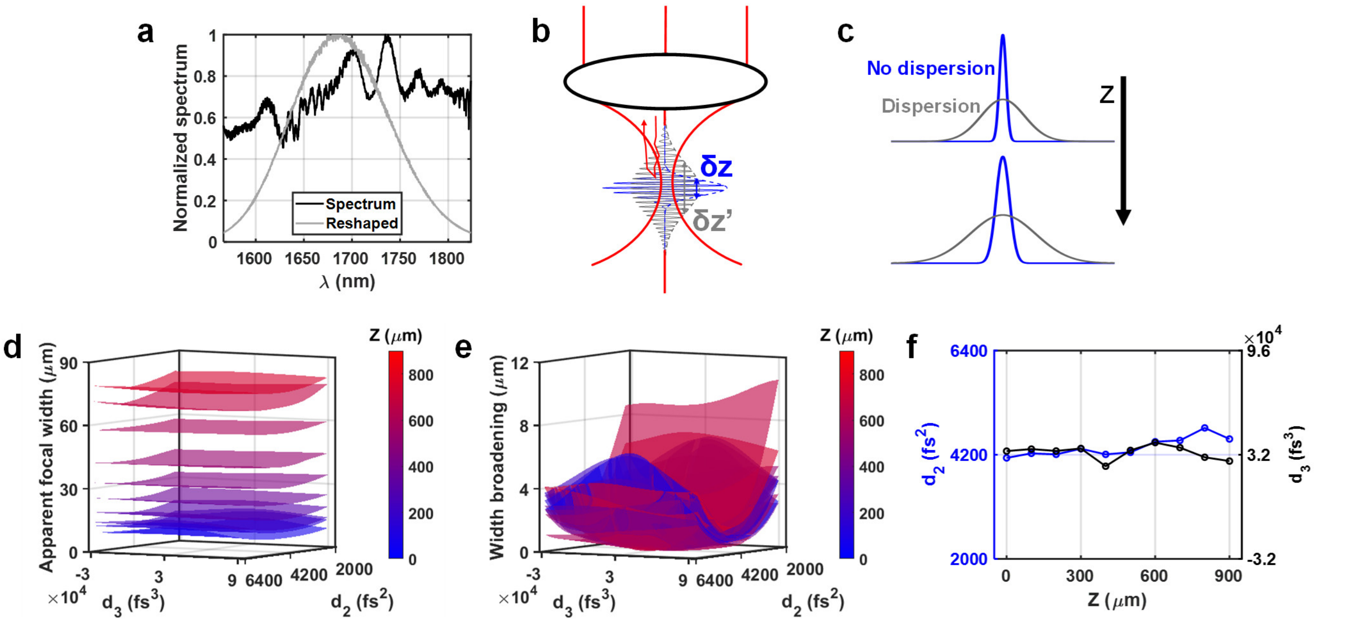

Fig. S1 Proposed method of numerical dispersion compensation in high numerical aperture (NA) OCM. a Registered reference spectrum (black). For mouse brain imaging, the spectrum is reshaped to be Gaussian (gray) with a 120 nm FWHM. **b** Chromatic dispersion induces a nonlinearity in the spectral phase, so the point spread function (PSF) is chirped and broadened (gray, $\delta z'$) compared to the ideal case with no dispersion (blue, δz). Multiply scattered paths (red) represent an additional source of broadening. **c** Thus, the width of the distribution of OCM depths (i.e., path length divided by 2) increases with focal depth (Z) due to multiple scattering (blue), and uncompensated dispersion results in further broadening (gray). The width of the distribution of OCM depths suggests optimal dispersion compensation coefficients, as seen from visualizations of the apparent focal width (**d**) and relative width broadening (**e**) at different focal depths. **f** The optimal second-order dispersion compensation coefficient (d_2) increases slightly with depth while changes in the third-order coefficient (d_3) are not detectable.

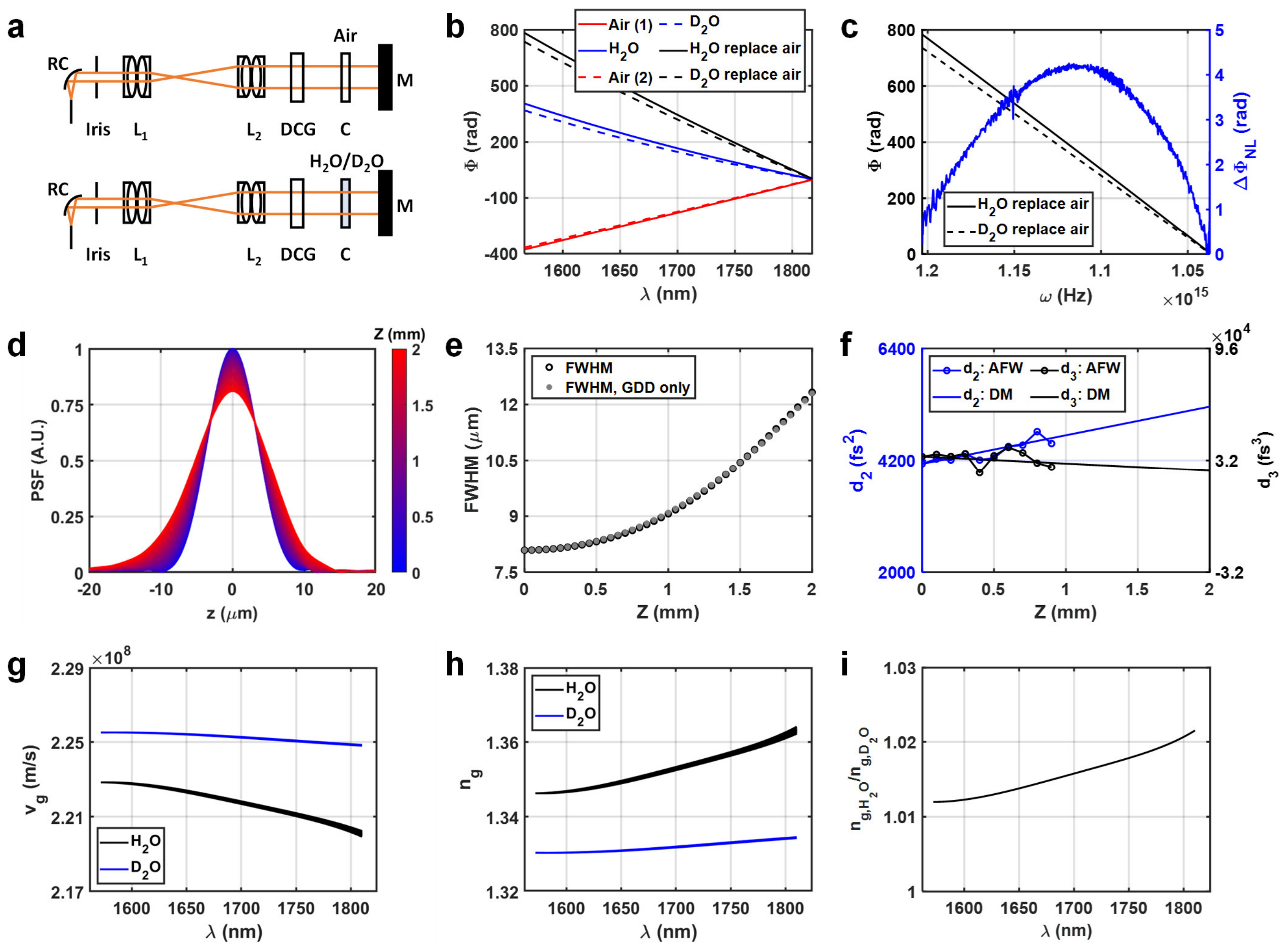

Fig. S2 Chromatic dispersion in the 1700 nm optical window. **a** Reference arm setup for dispersion measurements (sample arm not shown). The cuvette is either empty (filled with air, top panel) or filled with H_2O or D_2O (bottom panel). RC: reflective collimator; L_1, L_2: lenses (achromatic doublet pairs); DCG: dispersion compensation glass; C: cuvette; M: mirror. **b** Spectral phase (ϕ) of interferogram when a 2 mm cuvette in the reference arm is filled with H_2O (blue solid line), D_2O (blue dashed line) and air (red lines). Due to dispersion mismatch between arms, only phase changes between conditions are analyzed. Spectral phase changes when H_2O (black solid line) or D_2O (black dashed line) replaces 4 mm air (double pass path length), versus wavelength (**b**) and angular optical frequency (**c**), with the latter revealing a nonlinear spectral phase induced by replacing D_2O with H_2O [blue curve in (**c**)]. PSF axial profiles (**d**) and FWHMs (**e**) as H_2O replaces D_2O shows degradation of axial resolution up to 2 mm depth, as expected during deep focusing in OCM without compensating focus-dependent dispersion. GDD: group delay dispersion. **f** Optimized depth-dependent second (d_2) and third-order (d_3) dispersion compensation values obtained from dispersion measurement (DM) agree well with the apparent focal width (AFW) analysis (reproduced from **Fig. S1f**). Group velocity (**g**, v_g) and group refractive index (**h**, n_g) of H_2O (black) and D_2O (blue) obtained from spectral phase measurements. Calibrated limits of the system wavelength range are 1566.7 ± 2.1 and 1817.3 ± 2.8 nm. Shaded areas in (**g**)-(**h**) represent the range of solutions, accounting for wavelength calibration errors. **i** Group refractive index ratio of H_2O to D_2O.

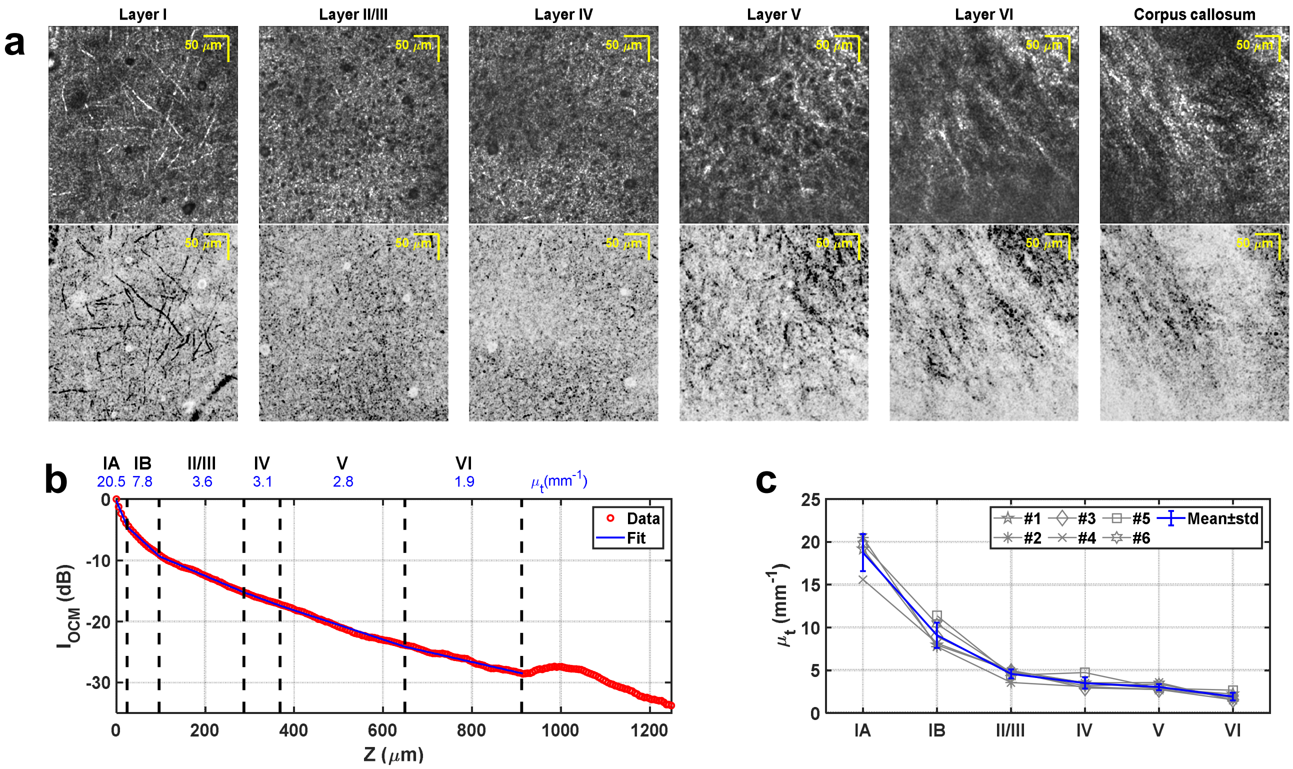

Fig. S3 Analysis of cortical lamination. a *En face* images of neuronal cell bodies (top row) and myelinated axons (inverted gray scale, bottom row) from the cortex and corpus callosum exhibit laminar trends of cytoarchitecture and myeloarchitecture, respectively. **b** Layer-by-layer attenuation coefficients are quantified with piecewise linear fitting (blue line) of background corrected OCM signal (red circles) versus depth. **c** Total attenuation coefficients of six animals (gray), with mean ± std (blue).

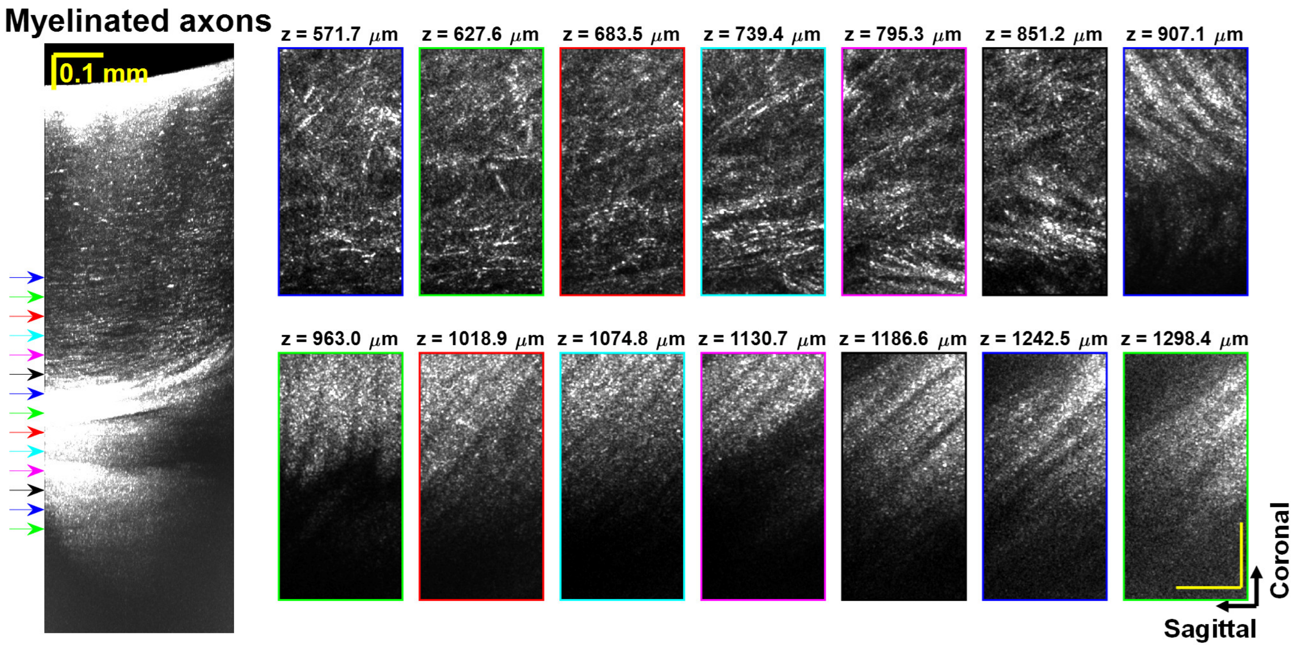

Fig. S4 *In vivo* visualization of myeloarchitecture. Outline colors of *en face* images correspond to arrow colors on the coronal image on the left, indicating projection locations. Axial projection depth: 11.2 µm. Coronal slice projection thickness: 190 µm. Scalebars represent 0.1 mm and apply to all the *en face* images.

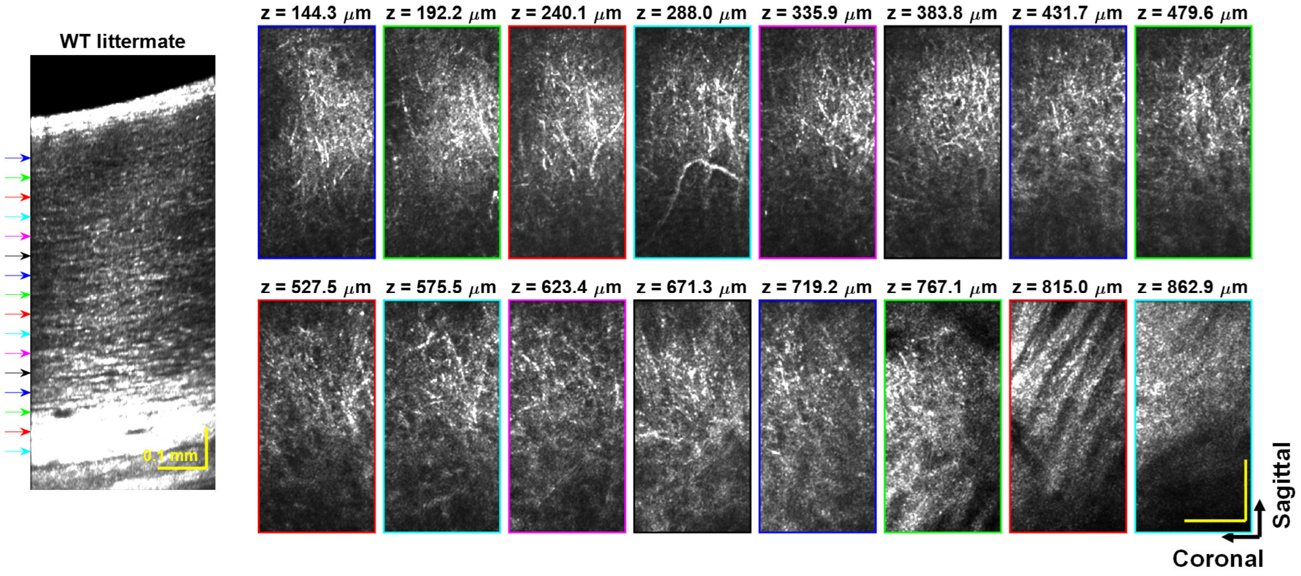

Fig. S5 3D biopsy of the WT littermate. Transverse images formed by processing to enhance plaques, similar to **Fig. 6e**. Outline colors of *en face* images correspond to arrow colors on the sagittal images on the left, indicating projection locations. Sagittal slice projection thickness: 17.8 μm. Axial projection depth: 16.0 μm. Scalebars represent 0.1 mm and apply to all the *en face* images.

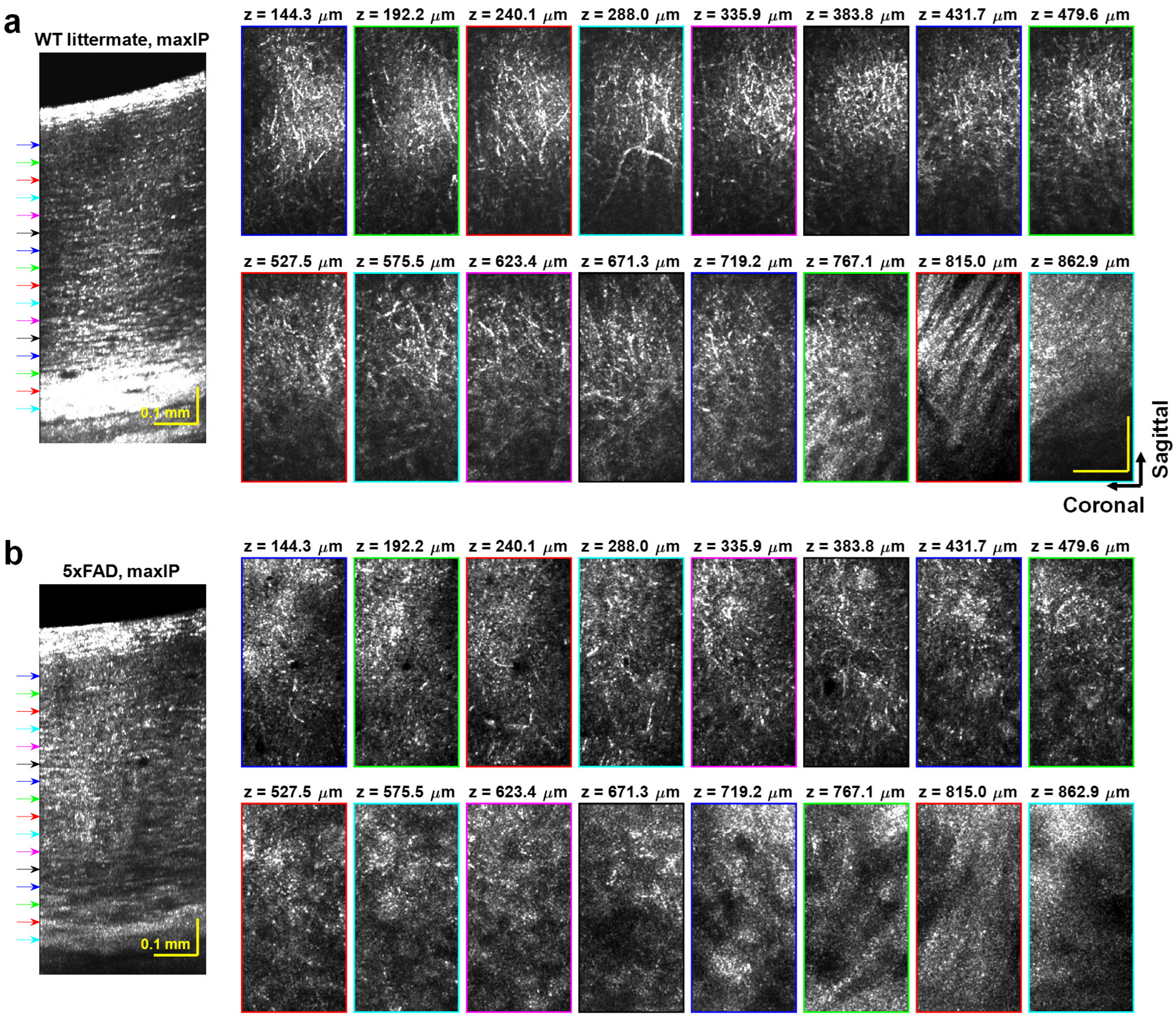

Fig. S6 Maximum intensity projection (maxIP) images of the WT littermate (a) and the 5xFAD mouse (b). Outline colors of *en face* images correspond to arrow colors on the sagittal images on the left, indicating projection locations. Sagittal slice projection thickness: 17.8 µm. Axial projection depth: 16.0 µm. Scalebars represent 0.1 mm and apply to all the *en face* images.

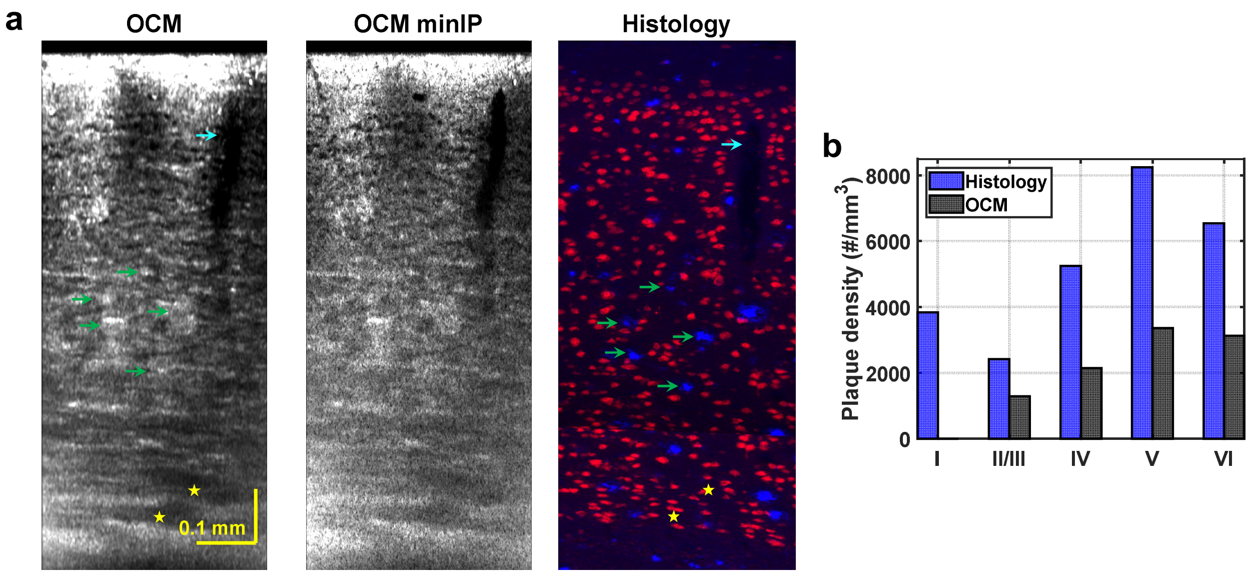

Fig. S7 Comparison of *in vivo* OCM imaging with *ex vivo* histology. a *In vivo* OCM imaging (left and middle) versus *ex vivo* histology (right). In the histological image, neuronal cell bodies are delineated by anti-NeuN staining (red), while amyloid plaques are highlighted by FSB staining (blue). Corresponding anatomical features include a blood vessel (cyan arrow), plaques (green arrows), and possible demyelination (yellow asterisks). OCM slice summation or minimum intensity projection (minIP) thickness: 17.8 µm. Histology slice thickness: 4 µm. Scalebars represent 0.1 mm and apply to all images. **b** Although differences are observed between OCM and histology, both modalities depict increasing plaque load in deep cortical layers.

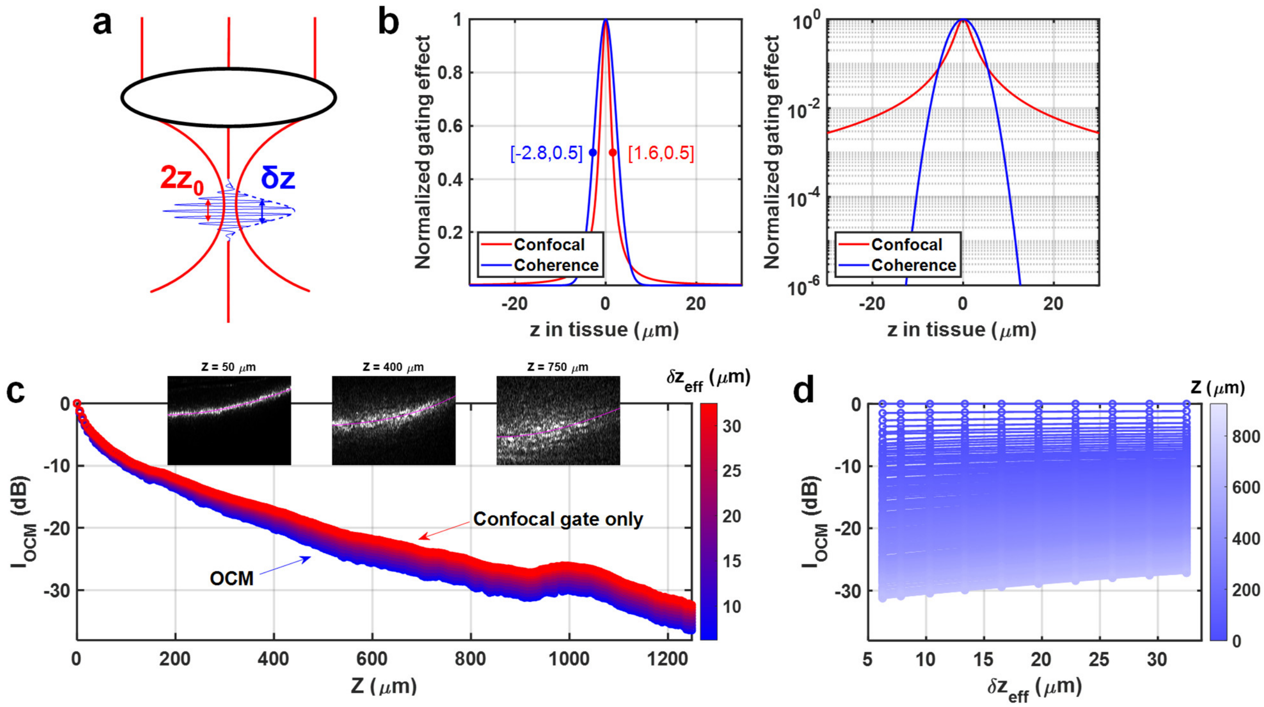

Fig. S8 Coherence gating complements confocal gating to reject multiply scattered light. a Coherence gate (blue, δz) and confocal gate (red, $2z_0$). **b** Intensity profiles of the two gates, shown in linear (left panel) and logarithmic (right panel) scales. **c-d** With a digitally-broadened coherence gate (δz_{eff}), the OCM signal decays slower with depth, suggesting inclusion of more multiply scattered light. Note that a large δz_{eff} compared to $2z_0$ results in only a confocal gate (red in **c**). Insets in (**c**) show broadening in OCM depth (proportional to path length) about the detected focus, suggesting that with a deep tissue focus, relatively more multiply scattered light passes the confocal gate.

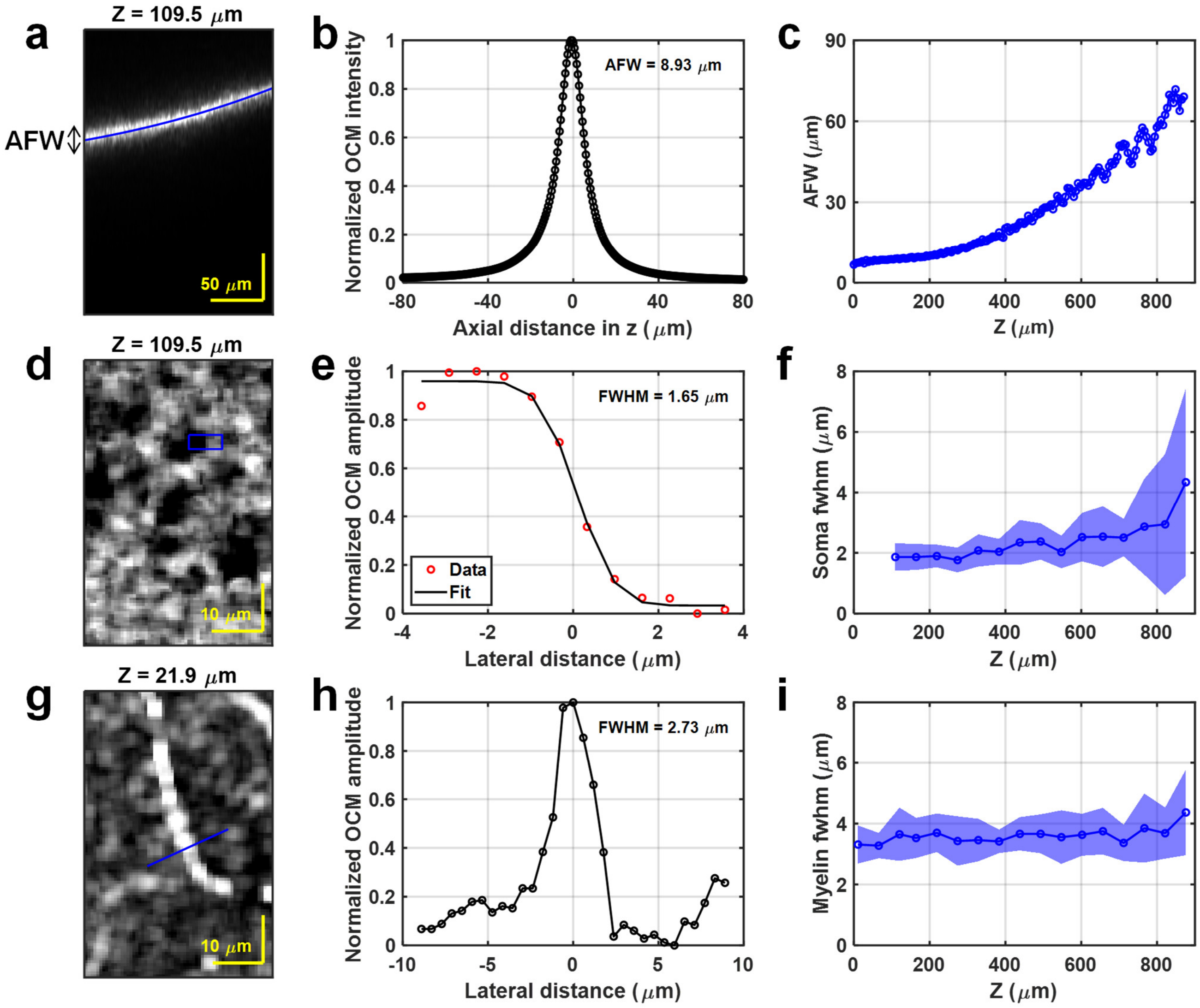

Fig. S9 Investigation of resolution degradation *in vivo*. Apparent focal width (AFW) (**a**-**c**), transverse FWHMs estimated from soma boundary profiles (**d-f**) and transverse FWHMs estimated from myelin profiles (**g-i**). The AFW (**a**), calculated similar to **Fig. S1d** based on the OCM intensity (**b**), shows a clear degradation with depth (**c**). To analyze the soma boundary, the amplitude in the blue boxed region in (**d**) is averaged along the vertical direction to generate the edge or step response (red circles, **e**), and then fitted with an error function (black line, **e**) to indirectly yield the lateral FWHM of the impulse response (**e**). The FWHM degradation with depth (**f**), with shaded regions representing standard deviations across 12 soma boundaries per depth, is subtle. To analyze myelin profiles, the amplitude profile perpendicular to the myelin axis (**g**) is calculated (**h**), and the lateral FWHM is determined directly (**h**). The degradation with depth (**i**), with shaded regions representing standard deviations across 92 axon cross sections per depth, is also subtle, consistent with (**f**).

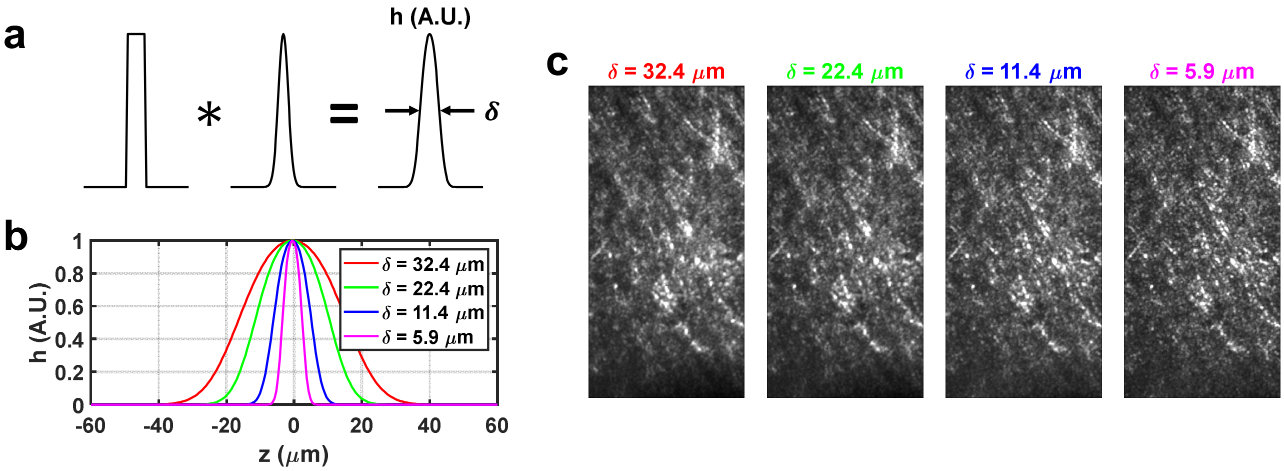

Fig. S10 Optimization of axial weighting function (*h*). **a** The shape of h is given by convolution of rectangular and Gaussian functions. **b** Axial weighting functions with different full-widths-at-half-maximum (FWHMs) (δ). **c** *En face* images at the same nominal cortical depth, derived from different weighting functions, exemplify the tradeoff between speckle reduction (large δ) and out-of-focus light suppression (small δ).
